# Glyphosate – Determination of glyphosate and AMPA in urine by LC-MS/MS: Biomonitoring Method – Translation of the German version from 2025

**DOI:** 10.34865/bi107183e10_1or

**Published:** 2025-03-31

**Authors:** Laura Kenny, Craig Sams, Kate Jones, Elisa Polledri, Rosa Mercadante, Silvia Fustinoni, Thomas Göen, Andrea Hartwig, MAK Commission

**Affiliations:** 1 Health and Safety Executive (HSE) Science and Research Centre Harpur Hill SK17 9JN Buxton (Derbyshire); 2 Laboratory of Environmental and Occupational Toxicology. Department of Clinical Sciences and Community Health. University of Milano and Fondazione IRCCS Ca Granda Ospedale Maggiore Policlinico Via Francesco Sforza 35 20122 Milano; 3 Friedrich-Alexander-Universität Erlangen-Nürnberg. Institute and Outpatient Clinic of Occupational, Social, and Environmental Medicine Henkestraße 9–11 91054 Erlangen Germany; 4 Institute of Applied Biosciences. Department of Food Chemistry and Toxicology. Karlsruhe Institute of Technology (KIT) Adenauerring 20a, Building 50.41 76131 Karlsruhe Germany; 5 Permanent Senate Commission for the Investigation of Health Hazards of Chemical Compounds in the Work Area. Deutsche Forschungsgemeinschaft, Kennedyallee 40, 53175 Bonn, Germany. Further information: Permanent Senate Commission for the Investigation of Health Hazards of Chemical Compounds in the Work Area | DFG

**Keywords:** glyphosate, AMPA, biomonitoring, urine, LC-MS/MS

## Abstract

The working group “Analyses in Biological Materials” of the German Senate Commission for the Investigation of Health Hazards of Chemical Compounds in the Work Area developed and verified the presented biomonitoring method. The aim of this method is the selective and sensitive quantitation of glyphosate (*N*-phosphonomethylglycine) and its only metabolite, aminomethylphosphonic acid (AMPA), in urine. Samples undergo solid-phase extraction prior to liquid chromatography-tandem mass spectrometry using glyphosate-2-^13^C,^15^N and AMPA-^13^C,^15^N,D_2_ as internal standards. Calibration is carried out with urine from persons with no known exposure to glyphosate and AMPA. The procedure has been comprehensively validated and the reliability data have been confirmed by replication and verification of the procedure in a second, independent laboratory. Good precision data with standard deviations of 1.3–9.8% for glyphosate and 1.9–5.4% for AMPA, as well as good accuracy data with mean relative recoveries in the range of 91–102% for glyphosate and 100–106% for AMPA, show that the method provides reliable and accurate analytical results. The method is both selective and sensitive, and the limits of quantitation of 0.1 μg/l for glyphosate and 0.5 μg/l for AMPA are sufficient to determine occupational exposure as well as some of the background exposure in the general population.

## 1 Characteristics of the method

**Table:** 

**Matrix**	Urine		
**Analytical principle**	Liquid chromatography with tandem mass spectrometry (LC‑MS/MS)		
**Parameters and corresponding hazardous substances**			
**Hazardous substance**	**CAS No.**	**Parameter**	**CAS No.**
Glyphosate (*N*‑Phosphonomethylglycine)	1071-83-6	Glyphosate; Aminomethylphosphonic acid (AMPA)	1071-83-6; 1066-51-9
Potassium glyphosate	70901-12-1; 39600-42-5		
Sodium glyphosate	34494-03-6		
Glyphosate sodium salt (2:3)	70393-85-0		
Ammonium glyphosate	40465-66-5		
Diammonium glyphosate	69254-40-6		
Triammonium glyphosate	114370-14-8		
Dimethylammonium glyphosate	34494-04-7		
Ethanolammonium glyphosate	40465-76-7		
Isopropylammonium glyphosate	38641-94-0		
Trimethylsulfonium glyphosate	81591-81-3		

### Reliability data

#### Glyphosate (method development using a ZORBAX Eclipse XDB‑C8 column)

**Table:** 

Within-day precision:	Standard deviation (rel.)	*s_w_* = 6.5% or 2.7%
	Prognostic range	*u* = 15.4% or 6.11%
	at a spiked concentration of 2 μg or 7 μg glyphosate per litre of urine and n = 8 or 10 determinations	
Day-to-day precision:	Standard deviation (rel.)	*s_w_* = 9.8%
	Prognostic range	*u* = 19.4%
	at a spiked concentration of 4 μg glyphosate per litre of urine and n = 381 determinations	
Accuracy:	Recovery (rel.)	*r* = 94% or 98%
	at a nominal concentration of 4 μg or 20 μg glyphosate per litre of urine and n = 5 determinations	
Limit of detection:	0.15 μg glyphosate per litre of urine
Limit of quantitation:	0.5 μg glyphosate per litre of urine

#### Glyphosate (external verification using a ZORBAX RR Eclipse XDB‑C8 column)

**Table:** 

Within-day precision:	Standard deviation (rel.)	*s_w_* = 2.4% or 2.2%
	Prognostic range	*u* = 6.7% or 6.1%
	at a spiked concentration of 2.5 μg or 25 μg glyphosate per litre of urine and n = 5 determinations	
Day-to-day precision:	Standard deviation (rel.)	*s_w_* = 3.4% or 2.4%
	Prognostic range	*u* = 14.6% or 10.3%
	at a spiked concentration of 2.5 μg or 25 μg glyphosate per litre of urine and n = 3 determinations	
Accuracy:	Recovery (rel.)	*r* = 99% or 100%
	at a nominal concentration of 2.5 μg or 25 μg glyphosate per litre of urine and n = 3 determinations	
Limit of detection:	0.1 μg glyphosate per litre of urine
Limit of quantitation:	0.5 μg glyphosate per litre of urine

#### AMPA (external verification using a ZORBAX RR Eclipse XDB‑C8 column)

**Table:** 

Within-day precision:	Standard deviation (rel.)	*s_w_* = 3.5% or 1.9%
	Prognostic range	*u* = 9.7% or 5.3%
	at a spiked concentration of 2.5 μg or 25 μg AMPA per litre of urine and n = 5 determinations	
Day-to-day precision:	Standard deviation (rel.)	*s_w_* = 5.4% or 2.6%
	Prognostic range	*u* = 23.2% or 11.2%
	at a spiked concentration of 2.5 μg or 25 μg AMPA per litre of urine and n = 3 determinations	
Accuracy:	Recovery (rel.)	*r* = 102% or 102%
	at a nominal concentration of 2.5 μg or 25 μg AMPA per litre of urine and n = 3 determinations	
Limit of detection:	0.1 μg AMPA per litre of urine
Limit of quantitation:	0.5 μg AMPA per litre of urine

#### Glyphosate (external verification using a Raptor Polar X column)

**Table:** 

Within-day precision:	Standard deviation (rel.)	*s_w_* = 2.1% or 1.3%
	Prognostic range	*u* = 5.8% or 3.6%
	at a spiked concentration of 2.5 μg or 25 μg glyphosate per litre of urine and n = 5 determinations	
Day-to-day precision:	Standard deviation (rel.)	*s_w_* = 4.3% or 2.1%
	Prognostic range	*u* = 18.5% or 9.0%
	at a spiked concentration of 2.5 μg or 25 μg glyphosate per litre of urine and n = 3 determinations	
Accuracy:	Recovery (rel.)	*r* = 102% or 100%
	at a nominal concentration of 2.5 μg or 25 μg glyphosate per litre of urine and n = 3 determinations	
Limit of detection:	0.05 μg glyphosate per litre of urine
Limit of quantitation:	0.1 μg glyphosate per litre of urine

#### AMPA (external verification using a Raptor Polar X column)

**Table:** 

Within-day precision:	Standard deviation (rel.)	*s_w_* = 2.8% or 3.8%
	Prognostic range	*u* = 7.8% or 10.5%
	at a spiked concentration of 2.5 μg or 25 μg AMPA per litre of urine and n = 5 determinations	
Day-to-day precision:	Standard deviation (rel.)	*s_w_* = 3.1% or 4.2%
	Prognostic range	*u* = 13.3% or 18.1%
	at a spiked concentration of 2.5 μg or 25 μg AMPA per litre of urine and n = 3 determinations	
Accuracy:	Recovery (rel.)	*r* = 106% or 100%
	at a nominal concentration of 2.5 μg or 25 μg AMPA per litre of urine and n = 3 determinations	
Limit of detection:	0.1 μg AMPA per litre of urine
Limit of quantitation:	0.5 μg AMPA per litre of urine

## 2 General information on glyphosate and AMPA

The substance *N*‑phosphonomethylglycine, better known as glyphosate, was developed by Swiss pharmaceutical company Cilag in the year 1950; it was later applied initially as a phosphonic acid-based water-softening agent (Székács and Darvas 2012). The U.S. company Monsanto recognised the potential of glyphosate for weed control and, in 1974, patented the substance as a broad-spectrum herbicide (Dill et al. 2010).

**Fig.1:**
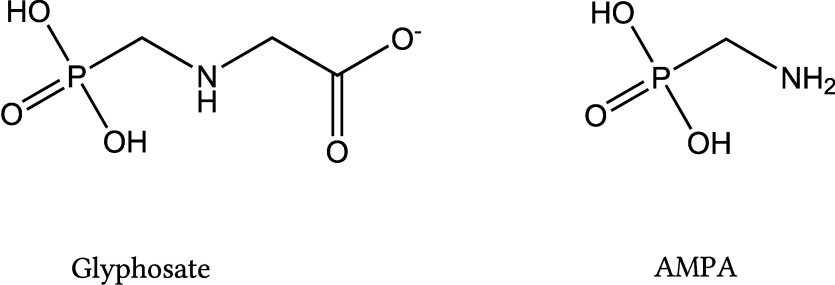
Structural formulas of glyphosate and AMPA

In its protonated form, pure glyphosate is a white, odourless solid (see Figure 1 for its structural formula), which is sparingly soluble in water (11.6 g/l at 25 °C) (IARC 2017). For this reason, glyphosate salts are usually used in herbicides, and various adjuvants are added to increase efficacy. Table 1 presents the most important glyphosate salts used as active ingredients in glyphosate formulations. Applied adjuvants primarily include surfactants, such as polyethoxylated tallow amine (Martins-Gomes et al. 2022), as well as antifoam agents, drift-control agents, or water-conditioning materials (Dill et al. 2010).

**Tab.1:** Glyphosate salts used as active substances in glyphosate formulations (according to ATSDR 2020)

Substance	CAS No.	Cation
Potassium glyphosate	70901-12-1; 39600-42-5	K^+^
Sodium glyphosate	34494-03-6	Na^+^
Glyphosate sodium salt (2:3)	70393-85-0	Na^+^
Ammonium glyphosate	40465-66-5	NH_4_^+^
Diammonium glyphosate	69254-40-6	NH_4_^+^
Triammonium glyphosate	114370-14-8	NH_4_^+^
Dimethylammonium glyphosate	34494-04-7	NH_2_(CH_3_)_2_^+^
Ethanolammonium glyphosate	40465-76-7	NH_3_(CH_2_CH_2_OH)^+^
Isopropylammonium glyphosate	38641-94-0	NH_3_CH(CH_3_)_2_^+^
Trimethylsulfonium glyphosate	81591-81-3	S(CH_3_)_3_^+^

In addition to Roundup^®^, there are currently more than 750 glyphosate-containing products available on the market; with an applied amount of about 800 000 t per year, this substance is the most widely used herbicide in the world (Antier et al. 2020; Polledri et al. 2023). The agricultural sector applies 90% of glyphosate-based herbicides, but these formulations are also used in landscaping, forestry, viticulture, railroad-track systems, and roadsides as well as in public parks and private gardens (Dill et al. 2010; Duke and Powles 2008; Jaworski 1972).

The herbicidal effect of glyphosate is based on the inhibition of 5‑enolpyruvylshikimate-3‑phosphate synthase (EPSPS), an enzyme which is only found in plants, fungi, and some microorganisms. In plants, EPSPS is essential for the bio­synthesis of aromatic amino acids, so that the uptake of glyphosate leads to an inhibition of protein biosynthesis, which is ultimately fatal for the plant (Dill et al. 2010; Jaworski 1972).

Studies in rats have shown that, with orally administered doses of 1–1000 mg glyphosate/kg body weight, about 20% is absorbed (EFSA 2023; EFSA et al. 2023). The absorbed glyphosate is distributed throughout the body, with the highest concentrations being found in bones, kidneys, and liver. In mammals, however, there is no indication of bioaccumulation. Glyphosate is primarily excreted within 48 hours: the unabsorbed glyphosate is excreted with the faeces, whereas the absorbed glyphosate is excreted predominantly unmetabolised with the urine (EFSA et al. 2023). Previous studies indicate that glyphosate is metabolised to a very limited extent in mammals, and that AMPA (see Figure 1 for its structural formula) is its only metabolite. In a study on twelve volunteers, Zoller et al. (2020) found that, 48 hours after consuming foods contaminated with glyphosate and AMPA, only 1% of the glyphosate dose was excreted with the urine, and the excreted AMPA concentrations corresponded to about 0.2% of the ingested amount of glyphosate. Based on current knowledge, glyphosate is eliminated biphasically, with half-lives of 2.1–7.5 h and 69–337 h (EFSA 2015 a). Assuming first-order kinetics, elimination half-lives of 5.5 h (Connolly et al. 2019), 4–17 h (Faniband et al. 2017), and 9 h (Zoller et al. 2020) have been reported for humans.

The main degradation product of glyphosate in the environment is AMPA. Glyphosate and AMPA can be detected in crops treated with glyphosate-containing herbicides as well as in surface waters, groundwater, and soil (Borggaard and Gimsing 2008). In the general population, exposure to glyphosate and AMPA can therefore originate from nutrition and the environment. It is generally assumed that only a small proportion of the AMPA detected in urine samples from the general population originates from the metabolic conversion of glyphosate and that the majority is ingested as part of the diet (EFSA 2015 a, b). Moreover, AMPA can be formed through the microbial and photochemical breakdown of aminophosphonic acids, which can be found in surfactants, flame retardants, and other industrial chemicals (Grandcoin et al. 2017; Struger et al. 2015). Since AMPA breaks down relatively slowly in the environment, it can accumulate in both plant and animal products (van Bruggen et al. 2018).

Background exposure to glyphosate and AMPA in the general population has been investigated in several biomonitoring studies. A general exposure was found, whereby mostly rather low levels in the range of the quantitation limit were determined. An overview of glyphosate and AMPA concentrations in urine samples from the German general population can be found in the method on the determination of glyphosate and AMPA in urine by GC‑MS/MS, which was published by the Commission in 2023 (Hoppe et al. 2023). A review paper by Gillezeau et al. (2019) summarised data on exposure to glyphosate and AMPA in general populations as well as in occupationally exposed workers from various countries. The glyphosate levels in urine samples from non-exposed individuals varied widely from region to region, averaging between 0.16 μg and 4 μg per litre of urine. In areas where glyphosate-containing herbicides were aerially applied, the average glyphosate concentration was 7.6 μg/l urine (Gillezeau et al. 2019; Varona et al. 2009).

Occupational exposure to glyphosate mainly occurs via inhalation and dermal routes. In a study on farmers, amenity horticulturalists, and forestry workers, Gillezeau et al. (2019) found average urinary glyphosate concentrations in the range of 0.26–73.5 μg/l, whereby the maximum exposure level measured was 233 μg/l in one U.S. farmer *(*Acquavella et al. 2004). In workers of a glyphosate factory in China, glyphosate concentrations of < 20–17 200 μg/l and AMPA concentrations of < 100–2730 μg/l were measured (Zhang et al. 2020). Glyphosate and AMPA concentrations in urine samples from workers of various industries, as well as the respective detection frequency and analytical method applied, can likewise be found in Hoppe et al. (2023).

In the European Union (EU), there have been three risk evaluations for glyphosate; in 2002, these assessments led to the initial approval of glyphosate in the EU. This approval was renewed in 2017 and, in 2023, the newest evaluation by the European Food Safety Authority (EFSA) and the European Chemicals Agency (ECHA) led to an extension of glyphosate approval in the EU until December 2033 (European Commission 2023). The International Agency for Research on Cancer (IARC) has classified glyphosate as probably carcinogenic to humans (Group 2A) (IARC 2017). In contrast, a 2022 risk assessment by ECHA concluded that glyphosate does not fulfil the scientific criteria for classification as a carcinogenic, germ-cell mutagenic, or teratogenic substance (RAC 2022). The possible carcinogenicity and other health risks (teratogenicity and endocrine disruption) of glyphosate are still a subject of debate (van Bruggen et al. 2018; Galli et al. 2024; US EPA 2017). Glyphosate has not yet been assessed by the Commission.

## 3 General principles

The analytical method described herein enables the quantitation of the herbicide glyphosate and its only metabolite, AMPA, in urine. After adding labelled internal standards (glyphosate‑2‑^13^C,^15^N; AMPA‑^13^C,^15^N,D_2_), the samples are prepared using solid-phase extraction. Analyte concentrations are determined using liquid chromatography with tandem mass-spectrometric detection. Calibration standards are prepared in urine obtained from persons with no known exposure to glyphosate or AMPA and processed in the same way as the samples to be analysed.

## 4 Equipment, chemicals, and solutions

### 4.1 Equipment

#### Method development

- Liquid chromatograph (e.g. Shimadzu LC‑20AB, Shimadzu UK Limited, Milton Keynes, United Kingdom) with a tandem mass spectrometer (e.g. AB SCIEX API 3200, AB SCIEX LLC, Framingham, MA, USA)
- Analytical column (e.g. ZORBAX Eclipse XDB‑C8, 4.6 × 150 mm, 5 μm, No. 993967-906, Agilent Technologies LDA UK Limited, Stockport, United Kingdom)
- C18 guard column (e.g. SecurityGuard Cartridges, AQ C18 4 × 2.0 mm, Nr. AJ0‑7510‑S, Phenomenex Ltd, Macclesfield, United Kingdom)
- Liquid-handling system (e.g. ASPEC^®^ GX‑271, No. 2614101, Gilson UK, Dunstable, United Kingdom)
- SPE cartridges (e.g. Strata SAX, 55 μm, 70 Å, 100 mg/1 ml, No. 8B‑S008‑EAK, Phenomenex Ltd, Macclesfield, United Kingdom)

#### External verification

- Liquid chromatograph (e.g. Agilent 1260 Infinity III LC System, Agilent Technologies Italia S.p.A., Cernusco sul Naviglio, Italy) with a tandem mass spectrometer (e.g. QTRAP^® ^5500 LC‑MS/MS system, AB SCIEX LLC, Framingham, MA, USA)
- Analytical column I (e.g. ZORBAX RR Eclipse XDB‑C8, 2.1 × 150 mm, 3.5 μm, No. 930990‑906, Agilent Technologies Italia S.p.A., Cernusco sul Naviglio, Italy)
- Analytical column II (e.g. Raptor Polar X, 50 × 2.1 mm, 2.7 μm, No. 9311A52, Restek S.r.l., Cernusco sul Naviglio, Italy)
- SPE cartridges (e.g. SampliQ Silica SAX, 500 mg/3 ml, No. 5982‑2035, Agilent Technologies Italia S.p.A., Cernusco sul Naviglio, Italy)

#### General equipment

- Laboratory centrifuge (e.g. Heraeus Deutschland GmbH & Co. KG, Hanau, Germany)
- Water-purification system (e.g. Milli‑Q^®^, Merck KGaA, Darmstadt, Germany)
- Heating block with nitrogen blower (e.g. Pierce Reacti‑Therm III 18935 Heating/Stirring Module with Reacti‑Vap III 18785 top unit, Pierce, Rockford, IL, USA)
- Analytical balance (e.g. Denver Instrument A‑200DS, No. 22827, American Laboratory Trading, East Lyme, CT, USA)
- 5‑ml, 25‑ml, 50‑ml, 150‑ml, 500‑ml, and 1000‑ml volumetric flasks (e.g. BRAND GMBH + CO KG, Wertheim, Germany)
- Variably adjustable pipettes, 10–100 μl and 100–1000 μl (e.g. Gilson UK, Dunstable, United Kingdom)
- Multipette^®^ with 20‑μl, 100‑μl, and 200‑μl Combitips^®^ (e.g. Eppendorf AG, Hamburg, Germany)
- Disposable 3.5-ml pipettes (e.g. Sarstedt AG & Co. KG, Nümbrecht, Germany)
- Graduated cylinder (e.g. BRAND GMBH + CO KG, Wertheim, Germany)
- Plastic vials with preformed polypropylene inserts and crimp caps (e.g. No. 24651, Restek GmbH, Bad Homburg vor der Höhe, Germany)
- 5‑ml polypropylene tubes, 10 × 75 mm (e.g. No 1E8Y.1, Carl Roth GmbH + Co. KG, Karlsruhe, Germany)
- 5‑ml polypropylene tubes, 12 × 55 mm (e.g. No. 115201, Greiner Bio-One International GmbH, Kremsmünster, Austria)
- Screw-top urine cups (e.g. Sarstedt AG & Co. KG, Nümbrecht, Germany)

### 4.2 Chemicals

Unless otherwise specified, all chemicals must be a minimum of *pro analysi* grade.

#### Method development

- Glyphosate PESTANAL^®^, ≥ 99% (e.g. No. 45521, Merck KGaA, Darmstadt, Germany)
- Glyphosate-2‑^13^C,^15^N (e.g. No. 90479, Merck KGaA, Darmstadt, Germany)
- Acetonitrile, HPLC grade (e.g. No. RH1015, Rathburn Chemicals Ltd., Walkerburn, United Kingdom)
- Formic acid (e.g. No. 5.43804, Merck KGaA, Darmstadt, Germany)
- Methanol, HPLC grade (e.g. No. RH1019, Rathburn Chemicals Ltd., Walkerburn, United Kingdom)
- Ultra-pure water (e.g. Milli‑Q^®^, Merck KGaA, Darmstadt, Germany)
- Nitrogen 5.0 (Air Liquide Deutschland GmbH, Düsseldorf, Germany)
- Urine from persons with no known exposure to glyphosate

#### External verification

- Aminomethylphosphonic acid (AMPA) ≥ 99% (e.g. No. 324817, Merck KGaA, Darmstadt, Germany)
- Aminomethylphosphonic acid‑^13^C,^15^N,D_2_, 100 μg/ml in water (e.g. No. CDNLM‑6786‑10, Cerilliant^®^, Merck KGaA, Darmstadt, Germany)
- Glyphosate PESTANAL^®^, ≥ 99% (e.g. No. 45521, Merck KGaA, Darmstadt, Germany)
- Glyphosate-2‑^13^C,^15^N (e.g. No. 90479, Merck KGaA, Darmstadt, Germany)
- Acetonitrile, HPLC grade (e.g. No. 34851, Merck KGaA, Darmstadt, Germany)
- Formic acid (e.g. No. 1.11670, Merck KGaA, Darmstadt, Germany)
- Methanol, HPLC grade (e.g. No. 34860‑R, Merck KGaA, Darmstadt, Germany)
- Ultra-pure water (e.g. Milli‑Q^®^, Merck KGaA, Darmstadt, Germany)
- Nitrogen 5.0 (Air Liquide Deutschland GmbH, Düsseldorf, Germany)
- Urine from persons with no known exposure to glyphosate or AMPA

### 4.3 Solutions

#### Method development (ZORBAX Eclipse XDB‑C8 column) and external verification (ZORBAX RR Eclipse XDB‑C8 column)

- 10% formic acid in methanol
  Some methanol is placed in a 150‑ml volumetric flask and 15 ml formic acid are added. The flask is then made up to the mark with methanol and the solution is thoroughly mixed.

The solution must be freshly prepared every workday.

- Eluent A (0.1% formic acid in water)
  Some ultra-pure water is placed in a 1000‑ml volumetric flask and 1 ml of formic acid is added. Subsequently, the flask is made up to the mark with ultra-pure water and the solution is mixed well.

The solution is stable for at least one week at room temperature.

#### External verification (Raptor Polar X column)

- 0.1% formic acid in water
  Some ultra-pure water is placed in a 1000‑ml volumetric flask and 1 ml of formic acid is added. Subsequently, the flask is made up to the mark with ultra-pure water and the solution is mixed well.

The solution is stable for at least one week at room temperature.

- Eluent A (0.5% formic acid in water)
  Some ultra-pure water is placed in a 1000‑ml volumetric flask and 5 ml of formic acid are added. Subsequently, the flask is made up to the mark with ultra-pure water and the solution is thoroughly mixed.

The solution is stable for at least one week at room temperature.

- Eluent B (0.5% formic acid in acetonitrile)
  Some acetonitrile is placed in a 1000‑ml volumetric flask and 5 ml of formic acid are added. Subsequently, the flask is made up to the mark with acetonitrile and the solution is thoroughly mixed.

The solution is stable for at least one week at room temperature.

### 4.4 Internal standards (ISTDs)

#### Method development

- Glyphosate-2‑^13^C,^15^N stock solution (200 mg/l)
  About 5 mg of glyphosate-2‑^13^C,^15^N are exactly weighed into a 25‑ml volumetric flask and dissolved in ultra-pure water. The volumetric flask is subsequently made up to the mark with ultra-pure water.
- Glyphosate-2‑^13^C,^15^N spiking solution (0.12 mg/l)
  In a 25‑ml volumetric flask, into which some ultra-pure water has been placed, 15 μl of the glyphosate‑2‑^13^C,^15^N stock solution are pipetted. The volumetric flask is subsequently made up to the mark with ultra-pure water.

The stock and spiking solutions are immediately transferred into plastic tubes and stored at –20 °C. Under these storage conditions, the analyte is stable for at least one year (Hoppe et al. 2023).

#### External verification

- Glyphosate-2‑^13^C,^15^N stock solution (100 mg/l)
  1 mg of glyphosate-2‑^13^C,^15^N is weighed into a 10‑ml volumetric flask and dissolved in ultra-pure water. The volumetric flask is subsequently made up to the mark with ultra-pure water.
- Glyphosate-2‑^13^C,^15^N and AMPA‑^13^C,^15^N,D_2_ spiking solution (2.5 mg/l each)
  125 μl of the glyphosate-2‑^13^C,^15^N stock solution and 125 μl AMPA‑^13^C,^15^N,D_2_ solution (100 mg/l) are pipetted into a 5-ml volumetric flask. The flask is subsequently made up to the mark with ultra-pure water.

The stock and spiking solutions are each immediately transferred into plastic tubes and stored at –20 °C. Under these storage conditions, the analytes are stable for at least one year (Hoppe et al. 2023).

### 4.5 Calibration standards

For the preparation of the calibration standards, urine from persons with no known exposure to glyphosate or AMPA is used. The calibration standards are prepared fresh for each analytical run and are processed in the same way as the samples to be analysed according to Section 5.2.

#### Method development

- Glyphosate stock solution (700 mg/l)
  17.5 mg glyphosate are weighed into a 25‑ml volumetric flask and dissolved in ultra-pure water. The volumetric flask is subsequently made up to the mark with ultra-pure water.
- Glyphosate working solution (0.4 mg/l)
  In a 50‑ml volumetric flask, 28.6 μl glyphosate stock solution are pipetted. The volumetric flask is subsequently made up to the mark with ultra-pure water.

The stock and working solutions are immediately transferred into plastic tubes and stored at –20 °C. Under these storage conditions, the analyte is stable for at least one year (Hoppe et al. 2023).

- Glyphosate spiking solution (20 μg/l)
  50 μl of the working solution are pipetted into a plastic tube into which 950 μl of urine from a person with no known glyphosate exposure has been placed.

The spiking solution must be freshly prepared every workday.

Calibration standards in a concentration range up to 20 μg/l are prepared in 5‑ml polypropylene tubes according to the pipetting scheme shown in Table 2.

**Tab.2:** Pipetting scheme for the preparation of calibration standards for the determination of glyphosate in urine (method development)

Calibration standard	Spiking solution [μl]	Urine [μl]	Glyphosate concentration [μg/l]
0	–	200	0
1	20	180	2
2	40	160	4
3	80	120	8
4	120	80	12
5	160	40	16
6	200	0	20

#### External verification

- Glyphosate stock solution (1000 mg/l)
  5 mg glyphosate are weighed into a 5‑ml volumetric flask and dissolved in ultra-pure water. The volumetric flask is subsequently made up to the mark with ultra-pure water.
- AMPA stock solution (1000 mg/l)
  5 mg AMPA are weighed into a 5‑ml volumetric flask and dissolved in ultra-pure water. The volumetric flask is subsequently made up to the mark with ultra-pure water.
- Working solution (100 mg/l)
  In a polypropylene tube, 400 μl of the glyphosate stock solution and 400 μl of the AMPA stock solution are added to 3200 μl of ultra-pure water by pipetting.
- Spiking solution I (250 μg/l)
  In a polypropylene tube, 10 μl of the working solution are added to 3390 μl ultra-pure water by pipetting.
- Spiking solution II (25 μg/l)
  500 μl of spiking solution I are placed in a 5‑ml volumetric flask by pipetting. The flask is then made up to the mark with ultra-pure water.
- Spiking solution III (2.5 μg/l)
  500 μl of spiking solution II are placed in a 5‑ml volumetric flask by pipetting. The flask is then made up to the mark with ultra-pure water.

For long-term storage, the stock, working, and spiking solutions of glyphosate and AMPA are immediately transferred into plastic tubes and frozen at –20 °C. Under these storage conditions, the analytes are stable for at least six months.

Calibration standards in urine are prepared in 5‑ml polypropylene tubes according to the pipetting scheme given in Table 3.

**Tab.3:** Pipetting scheme for the preparation of calibration standards for the determination of glyphosate and AMPA in urine (external verification)

Calibration standard	Spiking solution I [μl]	Spiking solution II [μl]	Spiking solution III [μl]	Urine [μl]	Glyphosate/AMPA concentration [μg/l]
0	–	–	–	1000	0
1	–	–	40	960	0.1
2	–	20	–	980	0.5
3	–	40	–	960	1
4	–	80	–	920	2
5	20	–	–	980	5
6	40	–	–	960	10
7	80	–	–	920	20
8	160	–	–	840	40

## 5 Specimen collection and sample preparation

### 5.1 Specimen collection

The urine samples are collected in suitable plastic cups and stored at −20 °C until processing.

### 5.2 Sample preparation

#### Method development

Prior to analysis, the urine samples are thawed at room temperature and thoroughly mixed. All samples are analysed in duplicate. A 200 μl aliquot is taken and pipetted into a polypropylene tube. Subsequently, 10 μl of the glyphosate‑2‑^13^C,^15^N spiking solution and 800 μl of ultra-pure water are added. The ASPEC^®^ system is loaded with SPE cartriges (Strata SAX, 100 mg/ml) and collection tubes, the samples are placed in the ASPEC^®^ rack and extracted as outlined below.

**Table:** 

Conditioning:	1 ml methanol
	1 ml ultra-pure water
Sample:	1 ml
Washing step:	1 ml ultra-pure water
	1 ml methanol
Drying step:	Air dry until the sample no longer drips
Elution step:	1 ml 10% formic acid in methanol

The eluate is evaporated to dryness under a gentle stream of nitrogen using a heating block set at 40 °C. The residue is reconstituted in 100 μl of 0.1% formic acid, mixed well, and transferred to the plastic insert of a LC injection vial. An aliquot of 10 μl is used for the analysis.

#### External verification

Prior to analysis, the urine samples are thawed at room temperature and thoroughly mixed. Of each urine sample, 1 ml are pipetted into a polypropylene tube. Subsequently, 10 μl of the glyphosate‑2‑^13^C,^15^N and AMPA‑^13^C,^15^N,D_2_ spiking solution are added. The samples are then purified using non-automated SPE. To this end, the SPE cartridges are conditioned using 2 ml of methanol, followed by 2 ml of ultra-pure water; after loading the sample, the cartridges are washed first with 2 ml of ultra-pure water, then with 2 ml of methanol and 1 ml of 10% formic acid in methanol. The analytes are eluted into polypropylene tubes using 1.5 ml of 10% formic acid in methanol.

The eluate is evaporated to dryness under a gentle stream of nitrogen in a heating block at 45 °C, and the residue is reconstituted in 100 μl of 0.1% formic acid in water. The reconstituted solution is thoroughly mixed and transferred into an LC injection vial with preformed plastic insert. An aliquot of 10 μl is applied for analysis.

## 6 Operational parameters

During method development, analysis was carried out on an LC‑MS/MS system comprised of a Shimadzu HPLC system and an AB SCIEX 3200 tandem mass spectrometer. The verifiers of the method used an Agilent 1260 HPLC system coupled with an AB SCIEX 5500 QTRAP^®^ tandem mass spectrometer.

The adjustments described in this section are instrument-specific and must be tested and adapted by the user as needed. The information given here is therefore only intended as a point of reference. It may be necessary to make further adjustments on instrumentations from other manufacturers.

### 6.1 High-performance liquid chromatography

#### Method development

**Table:** 

Analytical column:	ZORBAX Eclipse XDB‑C8, 4.6 × 150 mm, 5 μm
Separation principle:	Reversed phase
Eluent:	A: 0.1% formic acid in water
	B: acetonitrile
Injection volume:	10 μl
Flow rate:	0.4 ml/min
Gradient programme:	see Table 4

**Tab.4:** Gradient programme for the determination of glyphosate in urine (ZORBAX Eclipse XDB‑C8)

Time [min]	Eluent A [%]	Eluent B [%]
0	95	5
2	95	5
8	5	95
10	5	95
10.5	95	5
16	95	5

A six-port valve was used to direct the flow to waste and, at two minutes, to the ion source.

#### External verification I

**Table:** 

Analytical column:	ZORBAX RR Eclipse XDB‑C8, 2.1 × 150 mm, 3.5 μm
Separation principle:	Reversed phase
Column temperature:	40 °C
Eluent:	A: 0.1% formic acid in water
	B: acetonitrile
Injection volume:	10 μl
Flow rate:	0.2 ml/min
Gradient programme:	see Table 5

**Tab.5:** Gradient programme for the determination of glyphosate and AMPA in urine (ZORBAX RR Eclipse XDB‑C8)

Time [min]	Eluent A [%]	Eluent B [%]
0	100	0
2	100	0
8	5	95
10	5	95
11	100	0
20	100	0

#### External verification II

**Table:** 

Analytical column:	Raptor Polar X, 50 × 2.1 mm, 2.7 μm
Separation principle:	Hydrophilic interaction liquid chromatography (HILIC) and ion exchange
Column temperature:	40 °C
Eluent:	A: 0.5% formic acid in water
	B: 0.5% formic acid in acetonitrile
Injection volume:	10 μl
Flow rate:	0.5 ml/min
Gradient programme:	see Table 6

**Tab.6:** Gradient programme for the determination of glyphosate and AMPA in urine (Raptor Polar X)

Time [min]	Eluent A [%]	Eluent B [%]
0	35	65
1	35	65
4	90	10
9	90	10
10	35	65
15	35	65

### 6.2 Tandem mass spectrometry

The instrument-specific parameters must also be ascertained and adjusted by the user for the individual MS/MS system used. The instrument-specific parameters indicated below have been determined and optimised for the systems used during method development and external verification, respectively.

#### Method development

**Table:** 

Detection mode:	Multiple Reaction Monitoring (MRM)
Ionisation:	Electrospray, negative (ESI−)
Source temperature:	500 °C
Ion-spray voltage:	−4500 V
Curtain-gas flow:	50 l/min
Ion-source gas-1 flow:	70 l/min
Ion-source gas-2 flow:	50 l/min
CAD (charged aerosol detection):	High
Dwell time:	0.2 s
Parameter-specific settings:	see Table 7

**Tab.7:** Parameter-specific settings for the determination of glyphosate in urine (method development)

Substance	Retention time ZORBAX Eclipse XDB-C8 [min]	Precursor ion (***m/z***)	Product ion (***m/z***)
Glyphosate	3.5	168	63
Glyphosate-2‑^13^C,^15^N (ISTD)	3.5	170	63

#### External verification

**Table:** 

Detection mode:	Multiple Reaction Monitoring (MRM)
Ionisation:	Electrospray, negative (ESI−)
Source temperature:	600 °C
Ion-spray voltage:	−4500 V
Curtain-gas pressure:	20 psi (1.38 bar)
Gas 1 pressure:	80 psi (5.52 bar)
Gas 2 pressure:	60 psi (4.14 bar)
CAD (charged aerosol detection):	Low
Dwell time:	0.15 s
Entrance potential:	−10 V
Collision energy:	−20 V
Collision-cell exit potential:	−14 V
Parameter-specific settings:	see Table 8

**Tab.8:** Parameter-specific settings for the determination of glyphosate and AMPA in urine (external verification)

Substance	Retention time ZORBAX RR Eclipse XDB-C8 [min]	Retention time Raptor Polar X [min]	Precursor ion (***m/z***)	Product ion (***m/z***)
Glyphosate	1.87	10.87	168	63^a)^
		168	79^b)^
Glyphosate-2‑^13^C,^15^N (ISTD)	1.87	10.87	170	63
AMPA	2.02	1.44	110	63^a)^
		110	79^b)^
AMPA-^13^C,^15^N,D_2_ (ISTD)	2.02	1.44	114	63

^a)^ Quantifier

^b)^ Qualifier

## 7 Analytical determination

Of each sample prepared as described in Section 5.2, 10 μl are injected into the LC‑MS/MS system. Analytical separation is conducted by reversed-phase chromatography (ZORBAX Eclipse XDB‑C8 column or ZORBAX RR Eclipse XDB‑C8 column) or by HILIC and ion exchange (Raptor Polar X column). Identification of the analytes is based on their respective retention times and specific mass transitions. The retention times of the analytes and ISTDs listed in Tables 7 and 8 are intended only as a point of reference. Users must ensure the proper separation performance of the HPLC column used and the resulting retention behaviour of the analytes.

**Fig.2:**
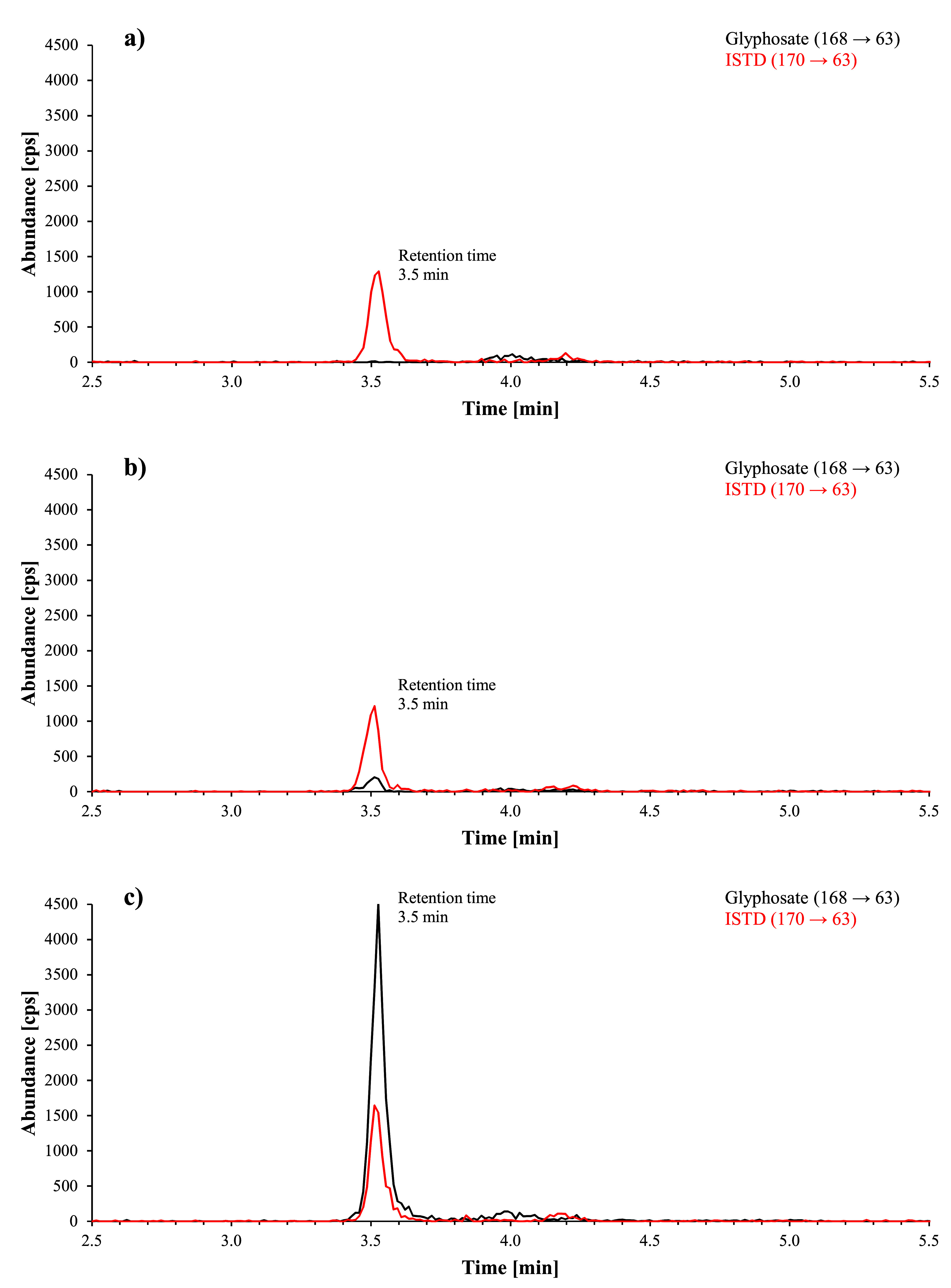
Chromatograms obtained during method development: a) of a urine sample from a person without known glyphosate exposure (glyphosate concentration < detection limit), b) of a native urine sample with a glyphosate background concentration of 1.2 μg/l and c) of a glyphosate calibration standard (20 μg/l urine)

**Fig.3:**
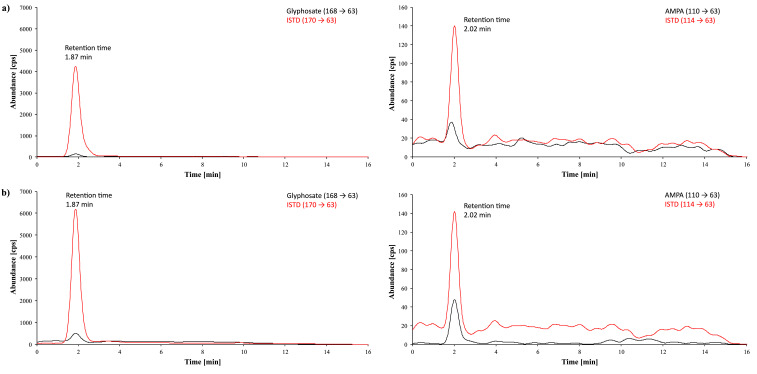
Chromatograms obtained during the external verification with the ZORBAX RR Eclipse XDB‑C8 column: a) a real sample with a glyphosate concentration of 1.4 μg/l urine and an AMPA concentration of 0.62 μg/l, b) a calibration standard with 1 μg glyphosate/l urine and 1 μg AMPA/l urine

**Fig.4:**
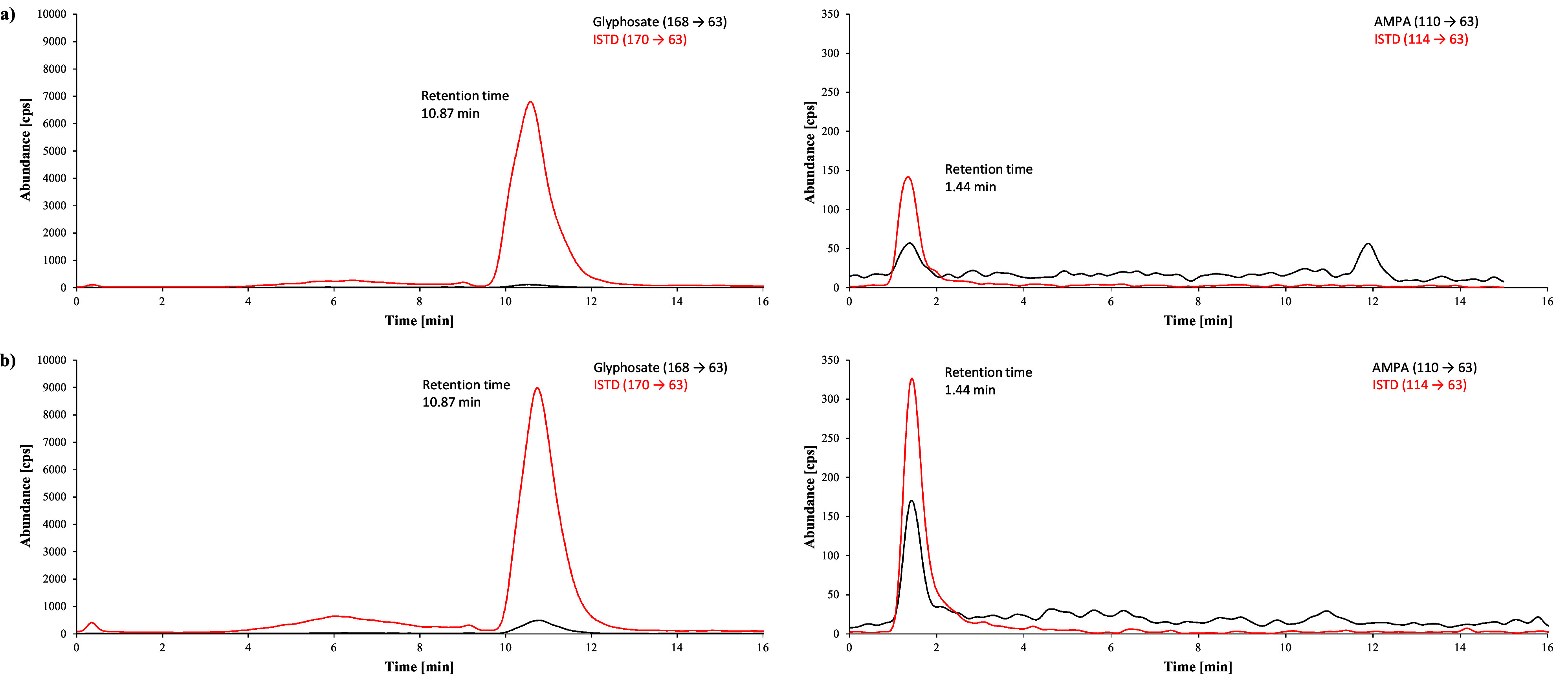
Chromatograms obtained during the external verification with the Raptor Polar X column: a) a real sample with a glyphosate concentration of 0.64 μg/l urine and an AMPA concentration of 0.62 μg/l urine, b) a calibration standard with 1 μg glyphosate/l urine and 1 μg AMPA/l urine

Figure 2 shows representative chromatograms obtained during method development of a urine sample from a person with no known glyphosate exposure, a native urine sample with a glyphosate background concentration of 1.2 μg/l and a calibration standard spiked with 20 μg glyphosate/l urine. The chromatograms of a real urine sample with a measured concentration of 1.4 μg glyphosate/l and 0.62 μg AMPA/l and of a calibration standard spiked with 1 μg glyphosate and 1 μg AMPA per litre of urine obtained during the external verification with the ZORBAX RR Eclipse XDB‑C8 column are shown in Figure 3. Figure 4 shows exemplary chromatograms obtained during external verification with the Raptor Polar X column for a native urine sample spiked with 0.64 μg glyphosate/l and 0.62 μg AMPA/l and a calibration standard spiked with 1 μg glyphosate and 1 μg AMPA per litre of urine.

## 8 Calibration

**Fig.5:**
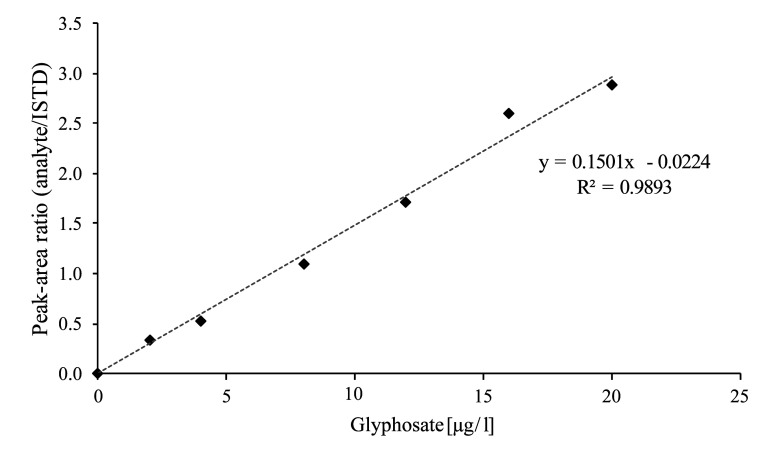
Representative calibration curve for the determination of glyphosate in urine (method development)

**Fig.6:**
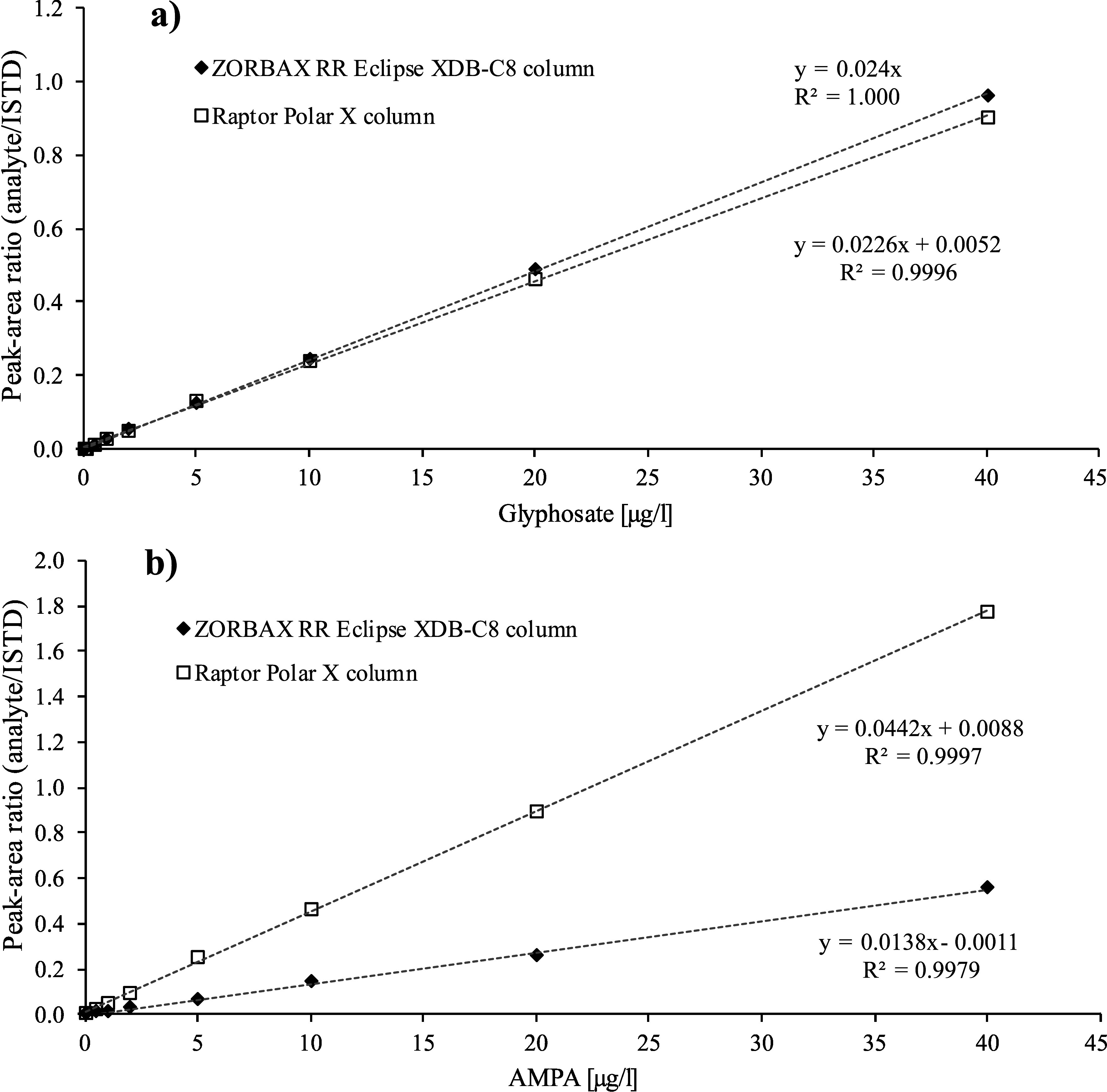
Representative calibration curves for the determination of glyphosate and AMPA in urine with the a) ZORBAX RR Eclipse XDB‑C8 column or the b) Raptor Polar X column, respectively (external verification)

The calibration standards described in Section 4.5 are processed in the same way as the samples (cf. Section 5.2) and analysed by LC‑MS/MS (cf. Section 6). Calibration curves are obtained by plotting the peak area ratios of the analyte and its corresponding ISTD against the spiked concentration of the respective calibration standard. The calibration curves are linear in the concentration range up to 20 μg glyphosate/l (method development) or up to 40 μg glyphosate/l or AMPA/l (external verification). As an example, Figure 5 shows a calibration curve generated by the developers of the method for the determination of glyphosate in urine. Figure 6 gives calibration curves for the determination of glyphosate and AMPA in urine, as generated during external verification using the ZORBAX RR Eclipse XDB‑C8 column and the Raptor Polar X column.

## 9 Calculation of the analytical results

The peak area of the analyte is divided by the peak area of the corresponding ISTD. The quotient thus obtained is entered into the calibration function (cf. Section 8), yielding the respective analyte concentration in μg/l. Because low levels of glyphosate and AMPA (mainly from dietary exposure) are present even in urine of unexposed persons, analyte peak areas are not corrected for blank values, and the slope of the calibration curve is used to calculate the concentration of unknown samples.

## 10 Standardisation and quality control

Quality assurance of the analytical results is carried out as stipulated in the guidelines of the *Bundesärzte­kammer* (German Medical Association) and in a general chapter published by the Commission (Bader et al. 2010; Bundes­ärztekammer 2014).

Material for quality control is not commercially available, such that a pooled-urine sample is spiked with a known amount of glyphosate and AMPA. The quality-control samples are–besides reagent blanks and urine blanks–included in each analytical run. The performance of the glyphosate and AMPA determination can also be monitored by participating in the G‑EQUAS external quality assessment scheme (https://www.g-equas.de/).

## 11 Evaluation of the method

The originally developed method enabled the quantification of glyphosate in urine; the glyphosate metabolite AMPA was not included in the method. The reliability of this method was confirmed by comprehensive validation as well as by replication and verification of the method in a second, independent laboratory.

In addition to replicating and validating the original method, the verifiers tested an alternative column and included the glyphosate metabolite AMPA. The following sections present both the validation data of the method originally developed for glyphosate and the data collected by the verifiers for glyphosate and AMPA.

### 11.1 Precision

#### Method development

Pooled urine spiked with 2 μg or 7 μg glyphosate/l was used to determine within-day precision. The spiked samples were processed (Section 5.2) and analysed (Section 6) in parallel as described. The data on within-day precision obtained by the developers of the method are shown in Table 9.

**Tab.9:** Within-day precision for the determination of glyphosate in urine (n = 8 (2 μg/l), n = 10 (7 μg/l)); method development

Analyte	Spiked concentration [μg/l]	Analytical column	Standard deviation (rel.) *s_w_* [%]	Prognostic range *u* [%]
Glyphosate	2	ZORBAX Eclipse XDB‑C8	6.5	15.4
	7		2.7	6.11

To determine day-to-day precision, urine spiked with 4 μg glyphosate/l was processed and analysed multiple times over four years. The precision data obtained by the developers are listed in Table 10.

**Tab.10:** Day-to-day precision for the determination of glyphosate in urine (n = 381); method development

Analyte	Spiked concentration [μg/l]	Analytical column	Standard deviation (rel.) *s_w_* [%]	Prognostic range *u* [%]
Glyphosate	4	ZORBAX Eclipse XDB‑C8	9.8	19.4

#### External verification

During external verification, the data for within-day precision and day-to-day precision were ascertained using a ZORBAX RR Eclipse XDB‑C8 column and a Raptor Polar X column, respectively. The urine used was spiked with 2.5 μg or 25 μg glyphosate and AMPA per litre. The data thus obtained are shown in Tables 11 and 12.

**Tab.11:** Within-day precision for the determination of glyphosate and AMPA in urine (n = 5); external verification

Analyte	Spiked concentration [μg/l]	Analytical column	Standard deviation (rel.) *s_w_* [%]	Prognostic range *u* [%]
Glyphosate	2.5	ZORBAX RR Eclipse XDB‑C8	2.4	6.7
	25		2.2	6.1
AMPA	2.5		3.5	9.7
	25		1.9	5.3
Glyphosate	2.5	Raptor Polar X	2.1	5.8
	25		1.3	3.6
AMPA	2.5		2.8	7.8
	25		3.8	10.5

**Tab.12:** Day-to-day precision for the determination of glyphosate and AMPA in urine (n = 3); external verification

Analyte	Spiked concentration [μg/l]	Analytical column	Standard deviation (rel.) *s_w_* [%]	Prognostic range *u* [%]
Glyphosate	2.5	ZORBAX RR Eclipse XDB‑C8	3.4	14.6
	25		2.4	10.3
AMPA	2.5		5.4	23.2
	25		2.6	11.2
Glyphosate	2.5	Raptor Polar X	4.3	18.5
	25		2.1	9.0
AMPA	2.5		3.1	13.3
	25		4.2	18.1

### 11.2 Accuracy

#### Method development

To determine the influence of urine matrix, the developers of the method analysed five individual urine samples (both unspiked and spiked with 4 μg or 20 μg glyphosate per litre). The results thus obtained for mean relative recovery are given in Table 13.

**Tab.13:** Mean relative recovery for the determination of glyphosate in individual urine samples (n = 5); method development

Analyte	Spiked concentration [μg/l]	Analytical column	Mean recovery (rel.) *r* [%]	Range [%]
Glyphosate	4	ZORBAX Eclipse XDB-C8	94	91–100
	20		98	95–102

#### External verification

During external verification, the accuracy for the determination of glyphosate and AMPA in urine was determined using the day-to-day precision data. The results obtained for the mean relative recovery are shown in Table 14.

**Tab.14:** Mean relative recovery for the determination of glyphosate and AMPA in urine (n = 3); external verification

Analyte	Spiked concentration [μg/l]	Analytical column	Mean recovery (rel.) *r* [%]	Range [%]
Glyphosate	2.5	ZORBAX RR Eclipse XDB-C8	99	96–103
	25		100	98–101
AMPA	2.5		102	97–107
	25		102	101–104
Glyphosate	2.5	Raptor Polar X	102	96–106
	25		100	99–102
AMPA	2.5		106	104–107
	25		100	97–105

### 11.3 Limits of detection and quantitation

The detection limit was determined on the basis of a signal-to-noise ratio of 3 ∶ 1 and the quantitation limit of a signal-to-noise ratio of 10 ∶ 1 (method development). The verifiers of the method calculated the limit of quantitation based on the blank value plus five times the standard deviation of the blank. The calculated values are listed in Table 15 (method development) and Table 16 (method verification).

#### Method development

**Tab.15:** Limits of detection and quantitation for the determination of glyphosate in urine; method development

Analyte	Analytical column	Detection limit [μg/l]	Quantitation limit [μg/l]
Glyphosate	ZORBAX Eclipse XDB-C8	0.15	0.5

#### External verification

**Tab.16:** Limits of detection and quantitation for the determination of glyphosate and AMPA; external verification

Analyte	Analytical column	Detection limit [μg/l]	Quantitation limit [μg/l]
Glyphosate	ZORBAX RR Eclipse XDB‑C8	0.1	0.5
AMPA	0.1	0.5
Glyphosate	Raptor Polar X	0.05	0.1
AMPA		0.1	0.5

### 11.4 Sources of error

The determination of glyphosate and AMPA in human urine is challenging and places high demands on the robustness of the LC‑MS/MS system. It is highly recommended to clean the device thoroughly between analytical series, especially when very large sample series have been measured.

In order to achieve the lowest possible quantitation limits for the determination of glyphosate and AMPA in urine, the selection of a suitable separation column is of utmost importance. The quantitation limits of 0.5 μg/l, as achieved with the RP columns (ZORBAX Eclipse XDB‑C8 and ZORBAX RR Eclipse XDB‑C8), are sufficient to measure both occupational exposure and high background levels in the general population. However, the Raptor Polar X column tested by the method verifiers, the separation principle of which is based on a combination of HILIC and ion exchange, yielded a quantitation limit for glyphosate that was five times lower. This finding could be primarily attributed to the considerably longer retention time for glyphosate on this column; by this, the matrix effects on the RP columns caused by non-retained salts and small molecules at retention times of less than 3.5 minutes were reduced.

It is known that glyphosate and AMPA may adsorb easily onto glass surfaces (Alferness et al. 2001). To avoid the adsorption of glyphosate and AMPA onto glass surfaces, urine samples and standard solutions must always be stored in plastic cups or tubes. Furthermore, when preparing solutions and calibration standards, analytes should always be pipetted into the respective solvent. However, Hoppe et al. (2023) also report potential interferences in the analysis of glyphosate and AMPA due to plastic additives found in polypropylene tubes.

For the purification and enrichment step by solid-phase extraction, the method verifiers used 10% formic acid for the final washing step as well as for the elution of the analytes. The method verifiers analysed each eluting fraction of the individual SPE steps and confirmed that no analyte losses could be observed when using 10% formic acid for the washing step, whereby the analytes are completely rinsed from the column during the elution step. The concentration of the sample solutions following SPE is not critical, as the highly polar analytes are not volatile.

## 12 Discussion of the method

The analytical method hereby presented was developed to quantify glyphosate in urine from workers (Connolly et al. 2018). The automated SPE used by the method developers allows for high sample throughput and is therefore very well-suited to routine analysis and large sample series. With a quantitation limit of 0.5 μg glyphosate per litre of urine, occupational exposures can be reliably measured (Connolly et al. 2019). The quantitation limit for glyphosate is comparable with those published by other working groups using LC methods: without derivatisation quantitation limits of 0.1–0.5 μg/l have been determined (Li and Kannan 2022; Nomura et al. 2020; Ruiz et al. 2021) and with derivatisation of 0.25–1 μg/l (Bienvenu et al. 2021; Bressán et al. 2021; Martin-Reina et al. 2021).

During external verification, in addition to glyphosate, its only metabolite, AMPA, was also integrated into the method. Since, with the LC-MS/MS system used by the method verifiers the same and a similar column as was used for method development led to considerably shorter retention times for glyphosate, other columns were tested (Polledri et al. 2023) and the method was also validated using an alternative column. This alternative column, the separation principle of which is based on a combination of HILIC and ion exchange, led to better separation of the analytes and to a considerably longer retention time for glyphosate (see Table 8).

The reliability criteria for the method are excellent for both glyphosate and AMPA. Accuracy was proven by good recovery after spiking pooled urine as well as individual urine samples.

Moreover, for glyphosate, the accuracy of the analytical results was proven by comparison with a GC method. To this end, 33 native urine samples were analysed using the presented LC‑MS/MS method (method development; Health and Safety Executive, Harpur Hill, Buxton, United Kingdom) as well as a GC‑MS/MS method (Hoppe et al. 2023) as part of an interlaboratory comparison. The glyphosate concentrations of 20 samples were found to be above the quantitation limits of both methods. The measurement results of these samples exhibit excellent correlation (see Hoppe et al. 2023). AMPA was not analysed as part of this interlaboratory comparison.

The sensitivity of the described method is sufficient to quantify occupational glyphosate exposure. Using a Raptor Polar X column, the calculated quantitation limits of 0.1 μg glyphosate/l urine and 0.5 μg AMPA/l urine are sufficient to measure higher levels of non-occupational exposure to glyphosate, e.g. via the diet.

**Instruments used during method development **LC‑MS/MS system: Shimadzu LC‑20AB (Shimadzu UK Limited, Milton Keynes, United Kingdom) with an AB SCIEX API 3200 tandem mass spectrometer (AB SCIEX LLC, Framingham, MA, USA); C18 guard column (Phenomenex Ltd, Macclesfield, United Kingdom); analytical column: ZORBAX Eclipse XDB‑C8, 4.6 × 150 mm, 5 μm (Agilent Technologies LDA UK Limited, Stockport, United Kingdom)

**Instruments used during external verification **LC‑MS/MS system: Agilent 1260 Infinity III (Agilent Technologies Italia S.p.A., Cernusco sul Naviglio, Italy) with a QTRAP^® ^5500 (AB SCIEX LLC, Framingham, MA, USA); analytical columns: ZORBAX RR Eclipse XDB‑C8, 2.1 × 150 mm, 3.5 μm (Agilent Technologies Italia S.p.A., Cernusco sul Naviglio, Italy) and Raptor Polar X, 50 × 2.1 mm, 2.7 μm, (Restek S.r.l., Cernusco sul Naviglio, Italy)
